# Tissue levels of suppressor of cytokine signaling-3 (SOCS-3) in mycosis fungoides

**DOI:** 10.1007/s00403-022-02339-x

**Published:** 2022-02-28

**Authors:** Hanan R. Nada, Laila A. Rashed, Ola Ouda Salman, Nermeen M. A. Abdallah, Mohamed M. Abdelhady

**Affiliations:** 1 Dermatology Department, Faculty of Medicine Kasr Alainy Hospitals, Cairo University, 174 Elyasmeen 2, 1st Settlement New Cairo, Cairo, Egypt; 2 Biochemistry Department, Faculty of Medicine/Cairo University, Cairo, Egypt; 3 Medical Microbiology and Immunology Department, Faculty of Medicine/Ain Shams University, Cairo, Egypt

**Keywords:** Mycosis fungoides, SOCS-3, JAK/STAT pathway

## Abstract

Mycosis fungoides (MF) is a type of cutaneous T-cell lymphoma with proposed multifactorial etiology. Suppressor of cytokine signaling-3 (SOCS-3) is one of the proteins expressed in MF. Its exact role in disease pathogenesis has not yet been thoroughly investigated. This study aimed to assess the expression of SOCS-3 in patients’ skin with mycosis fungoides to elucidate their possible role in the pathogenesis in MF. 30 patients with mycosis fungoides and 30 age and sex-matched healthy controls were included. After clinical examination, tissue levels of SOCS-3 were measured by ELISA. The level of expression of SOCS-3 was significantly upregulated in the lesional tissue compared to perilesional SOCS-3 level in patients’ group (*P* < 0.001), and both levels were higher than the SOCS-3 level in control group (*P* < 0.001). In addition, there was a statistically significant positive correlation between lesional SOCS-3 level and itching in patients’ group (*P* < 0.001). Regarding lesional and perilesional SOCS-3 levels in each stage, there was a significant increase in lesional SOCS-3 levels in comparison to perilesional level whether in stage Ia, Ib, and IIa; (*P* < 0.001), (*P* < 0.001) and (*P* < 0.001), respectively. Increased tissue levels of SOCS-3 patients with mycosis fungoides point to a role that SOCS-3 could play in its pathogenesis. Also, high levels of SOCS-3 in MF patients with itching suggest a role in the pathogenesis of this symptom. These findings may prove helpful in formulating a new treatment modality in addition to the current treatment of MF.

## Introduction

Suppressors of cytokine signaling (SOCS) are regulator proteins that inhibit Janus Kinases‐Signal Transducer and Activator of Transcription (JAK-STAT) signaling pathways linked to type I and II cytokine receptors. Eight proteins are recognized: SOCS1 through 7 and the cytokine-inducible SH2 domain-containing protein (CIS) [1].

SOCS-3 can control both innate and acquired immune responses by regulating cytokine-mediated inflammatory signals, e.g., IL-6, IL-10 families, IFN-α, IFN-γ and growth hormone. SOCS proteins also can be regulated by cytokines, besides Toll-like receptors ligands (Table 1) [1–4].

**Table 1:** Role of SOCS3 in immune modulaton

Immune cell	Role of SOCS3 in immune modulation
Dendritic cell (DC)	Through inhibition of STAT6, SOCS3 promotes DC maturation Moreover, SOCS3 is likely to act as both a promoter and an inhibitor of DC function depending on the type of enzyme regulated i.e. SOCS3 promotes indoleamine 2,3-dioxygenase degradation and enhances DCs antitumor effects. But on binding to kinase type M2 enzyme on DCs reduces the ATP production and weakens the curative effect of antitumor immune therapy, which indicates that SOCS3 manipulates the activation and function of DCs in the tumor microenvironment [3]
Macrophages (MQs)	Without SOCS3, MQs are less likely to develop pro-inflammatory characteristics and instead exhibit immunoregulatory traits. In MQs, a lack of SOCS3 inhibits M1 activation and promotes anti-inflammatory responses. SOCS3 deficiency in MQs, on the other hand, suppresses tumor growth and prevents cancer metastasis, which is linked to elevated M1 cytokines levels [4]
T cells	SOCS3 inhibits IL12-mediated STAT4 activation, which promotes Th2 differentiation and inhibits Th1 differentiation SOCS3 is a major negative regulator of STAT3 phosphorylation and Th17 generation induced by IL-23 Tregs are deficient in SOCS3 protein expression. And over expression of SOCS3 decreases foxp3 expression and Tregs [1]

Diminished expression of SOCS-3 is linked to various autoimmune diseases (e.g., rheumatoid arthritis and multiple sclerosis), while high expression is related to some metabolic diseases and immune evasion of certain bacterial and viral infections [5–7].

Suppressor of cytokine signaling 3 can act either as an oncogene or a tumor suppressor, depending on cellular context. SOCS3 regulates numerous tumors through inhibition of various signaling pathways and functions as a tumor suppressor gene (Table 2). The expression and function of SOCS3 vary significantly among different tumor types [8–11]. SOCS3 also promotes melanoma progression and attenuates the therapeutic efficacy of IFN-α and IFN-γ treatments [12]. Therefore, a comprehensive understanding of the functions and mechanisms regulating the expression of SOCS3 might facilitate their future clinical applications, either as diagnostic or prognostic biomarkers [1].

**Table 2:** Role of SOCS-3 in different tumors

Genes	Cell types	Data sources	Expression	Function	Regulated target genes
SOCS-3	Breast cancer cell	Cell lines Mouse xenograft model	Downregulation Upregulation	Upregulate inflammatory cytokine IL-6 Inhibit tumor growth and reduce circulating tumor cells	IL-6/STAT3/NF-κB [8]
	Prostate cancer cell	Cell lines	Upregulation	Inhibit androgen-mediated proliferation and secretion	CDK2, CDK4, cyclins E, and D1 [9]
	Melanoma cell	Cell lines	Upregulation	Influence the responsiveness of melanoma cells to IFN-a and IFN-γ	STAT1, ISG-15, OAS1, and IRF1 [12]
	Malignant pleural mesothelioma cell	Cell lines Pleural xenograft model	Upregulation	Induce apoptosis and partial G0/G1 arrest and inhibit tumor growth	JAK/STAT3, ERK, FAK, and p53 [10]
	Head and neck squamous cell carcinoma cell	Human samples Cell lines	Downregulation Upregulation	Cause growth inhibition and apoptosis	STAT3, Bcl-2, Bcl-xL [11]

Regarding skin tumors, basal cell carcinomas and squamous-cell carcinomas exhibit diminished expression of SOCS-3 compared to any other inflammatory skin condition [11, 13]. On the other hand, Cutaneous T-Cell Lymphoma (CTCL) and melanoma cells express a high level of SOCS-3, which protects malignant cells from cytokine-mediated antitumor effect, e.g., IFN-α [7, 14, 15].

Mycosis Fungoides (MF) is considered a low-grade cutaneous lymphoma with CD4 helper T cell phenotype, accounting for most CTCLs. The first cutaneous manifestation of MF is nonspecific flat erythematous macules (the patch stage) that may progress to infiltrating plaque (plaque stage) and eventually in some patients, nodules, and tumors (tumor stage) [16].

It was estimated that the prevalence of early stage MF is 4.8-6.6/100,000 in the United Kingdom and United States [17]. Transition to advanced disease is characterized by a shift from the antitumor response of immune cells toward inflammatory microenvironment that facilitates the expansion of malignant T cell with an observed shift from TH1 response toward TH2 dominant environment, aiding immune evasion of MF [15].

Abnormal expression of SOCSs has been discovered in cancer cells and immune cells in the tumor microenvironment. Downregulating the expression of SOCS proteins in immunocytes, such as DCs and T cells, enhances the antitumor immunity by increasing STATs activation [18]. The infection of cells with oncolytic adenovirus CN305 (AdCN305)-SOCS3 and AdCN305 cell-penetrating peptides-SOCS3 (membrane permeable SOCS3) result in considerable cytotoxicity of liver tumor cells and downregulation of cyclin D1 and Bcl-xL [19]. Tyrosine kinase inhibitor peptide, Tkip, was developed as a mimetic of SOCS proteins and effectively inhibited the JAK2-mediated phosphorylation of STAT1 and proliferation of prostate cancer cells [20]. Platelet factor-4 enhances the expression of SOCS3 protein, thereby suppressing the STAT3 activation and inducing cell apoptosis in myeloma [21]**.** Therefore, studies on the SOCS family will help understand the molecular mechanisms of SOCS-mediated tumor progression and help oncologists determine appropriate therapeutic options [14].

The study aimed to assess the expression of suppressor of cytokine signaling-3 (SOCS-3) in patients’ skin with mycosis fungoides to evaluate the possible role of SOCS-3 expression in the pathogenesis of mycosis fungoides.

## Materials and methods

This case–control study was conducted at the Dermatology outpatient clinic, Kasr El-Ainy Hospital, Faculty of Medicine, Cairo University, after approval of the Dermatology Research Ethical Committee (Derma REC). Written informed consents were obtained from all patients and controls. The study was conducted on 30 patients with proven histopathological Mycosis Fungoides (MF) patch stages (Ia, Ib, IIa) and 30 sex, age-matched healthy controls from February 2019 to November 2019.

Patients were excluded from the study if they were on topical or systemic treatment at least 4 weeks before the study, or on systemic retinoids, or have diseases or cancers pathology known to involve dysregulation of SOCS-3 as autoimmune disease (diabetes mellitus type-1, systemic lupus erythematosus, rheumatoid arthritis, multiple sclerosis, psoriasis), or cancers known because of SOCS-3 dysregulation as (barret adenocarcinoma, liver cancer, lung cancer, prostate cancer, melanoma, head and neck squamous cell carcinomas) or patients with hepatitis-C virus.

All the patients were subjected to the following:

- 1. Detailed history taking including onset, course, duration of disease, history of itching (Assessment of degree of itching was done by verbal rating scale (no itching = 0, Mild = 1, Severe = 2), presence of any medical disorders, e.g., diabetes and hypertension.
- 2. Clinical assessment was done to exclude any other associated dermatologic diseases and to determine the extent of MF, degree of erythema, scales, and lymph node examination. Extent was estimated using the role of palm, patient’s hands considered as (1%).
- 3. Investigations.

Skin biopsy.

Local anesthetic Lidocaine was injected in the area to be biopsied. Two biopsies were taken—the first one (2 mm) punch biopsy from perilesional skin (2 cm away from the lesion). The second biopsy (4 mm) punch biopsy from lesional skin was cut into two halves; one half was preserved in formalin for Hematoxylin and Eosin staining for standard histopathological examination, while the other half was preserved in Phosphate Buffer Saline (PBS) for ELISA.

In the control group, a 4 mm punch biopsy was taken from the normal skin of each subject. All biopsies for ELISA testing were preserved in phosphate buffer saline (PBS) and kept frozen at − 80 °C.

SOCS-3 tissue levels by ELISA.

Tissues were minced and rinsed in ice-cold PBS and then homogenized in PBS followed by centrifugation for 5 min at 5000 × *g* to get the supernatant. SOCS-3 was measured in the supernatant using Human SOCS-3 ELISA Kit (catalog no. MBS2508282; MyBioSource, San Diego, CA, USA) according to the manufacturer's protocol. The optical density (OD) was measured by a Microplate reader at a wavelength of 450 nm.

- Statistical methods

Data were coded and entered using the statistical package for the Social Sciences (SPSS) version 25 (IBM Corp., Armonk, NY, USA). Data were summarized using mean, standard deviation, minimum and maximum for quantitative variables and frequencies (number of cases), and relative frequencies (percentages) for categorical variables. For comparing categorical data, chi-squared (*χ*^2^) test was performed. An exact test was used instead when the expected frequency was less than 5. The correlation between quantitative variables was done using the Spearman correlation coefficient, and *P* values less than 0.05 were considered statistically significant.

## Results

This study cohorts included 30 patients with patch stage mycosis fungoides [Ia were 7 (23.3%), Ib were 16 (53.3%) while IIa were 7 (23.3%)], and 30 age and sex-matched (*P* value > 0.05), healthy subjects who served as controls.

The disease duration ranged between 2.00 and 24.00 years with a mean of 9.50 ± 5.78. Regarding the body surface area involved, it ranged between 6.00 and 95.00 with a mean of 38.92 ± 29.52.

**Fig. 1:**
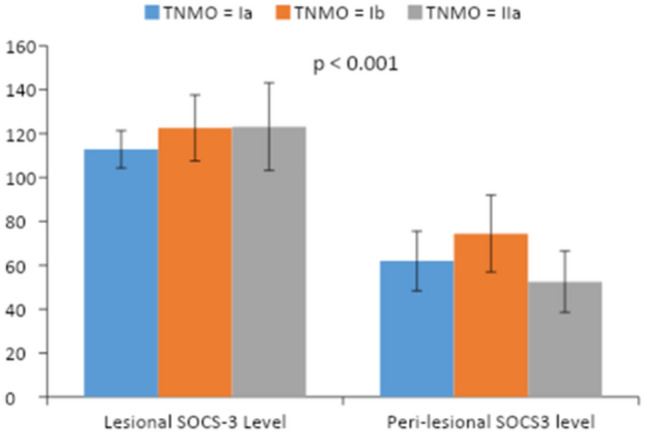
Patients group regarding the degree of itching, erythema, and scales

The degree of erythema of skin lesions ranged between 0.00 (no erythema) and 3.00 (severe erythema) with a mean of 1.13 ± 0.78, scaling ranged between 0.00 (no scales) and 2.00 (severe scales) with a mean of 1.17 ± 0.53, while the degree of itching ranged between 0.00 (no itching) and 3.00 (severe itching) with a mean of 0.83 ± 0.95. (Fig. 1).

### Levels of SOCS-3 in patients and control group

The levels of SOCS-3 in patients and control are shown in Table 3. The mean value in skin lesion was higher than that of perilesional skin, which was higher than that of controls. There was a statistically significant difference between the mean values of SOCS-3 in both lesional skin, perilesional skin in patients and controls (*P* < 0.001). As well as a significant difference between lesional and perilesional levels of SOCS-3 (*P* value < 0.001) with higher expression in lesional skin than that of perilesional skin.

**Table 3:** Levels of SOCS-3 in patients and controls

Variable		Controls (*n* = 30)	Patients (*n* = 30)		*P value
			Peri-lesional skin	Lesional skin	
Level of SOCS-3	Range	31.00–62.00	36.90–101.60	87.60–147.10	
	Mean ± SD	40.72 ± 7.80	66.42 ± 17.98	120.44 ± 15.24	< 0.001

*P value for SOCS-3 level in control vs lesional and vs perilesional skin area, and for lesional vs perilesional skin area

### Comparison between lesional and perilesional SOCS-3 Levels

**Fig. 2:**
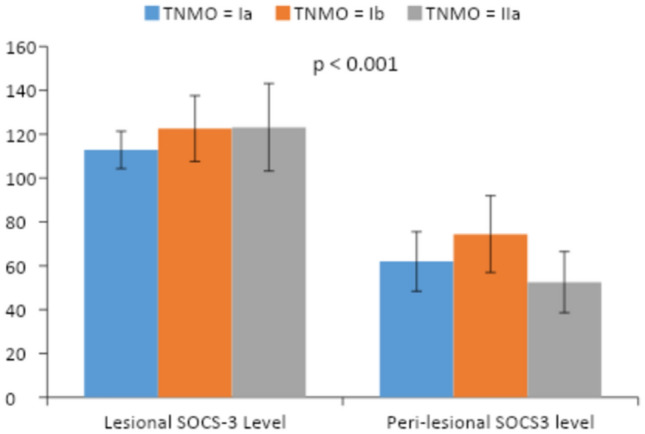
Bar chart showing a comparison between lesional and perilesional SOCS-3 Levels in stage Ia, Ib, and IIa in patients' group

In the patients’ group, there is a significant difference between lesional and perilesional SOCS-3 Levels in stage Ia, Ib, and IIa with higher expression of SOCS-3 in lesional skin than that in perilesional, *P* value < 0.001, < 0.001, < 0.001 respectively (Table 4) (Fig. 2).

**Table 4:** Comparison between lesional and perilesional SOCS-3 levels in each stage

Variable		Range	Mean ± SD	*P* value
Ia	Peri- lesional SOCS-3	36.90–76.30	61.96 ± 13.57	< 0.001
	Lesional SOCS-3	102.10–126.30	112.83 ± 8.52	
Ib	Peri-lesional SOCS-3	53.10–101.60	74.45 ± 17.49	< 0.001
	Lesional SOCS-3	103.50–147.10	122.58 ± 15.01	
IIa	Peri-lesional SOCS-3	37.80–72.30	52.53 ± 13.93	< 0.001
	Lesional SOCS-3	87.60–145.20	123.17 ± 19.96

### Correlations of SOCS-3 level with demographic and clinical variables in patients

There was no statistical correlation between SOCS-3, demographic and clinical data of the patients regarding their age, sex, duration, BSA, erythema, scaling in lesional and perilesional skin. There was only a significant positive correlation between SOCS-3 and itching in lesional skin (*P* = 0.016) (Table 5).

**Table 5:** Correlation between lesional and perilesional SOCS-3 and different studied variables in the patients’ group

Variables		Lesional SOCS-3		Peri-lesional SOCS-3	
		*R*	*P* value	*R*	*P* value
Sex	Male (*n* = 10)	**_**	0.212	**_**	0.780
	Female (*n* = 20)				
Age		-0.061-	0.748	0.034	0.859
Duration (y)		0.065	0.734	-0.056-	0.768
BSA		0.227	0.228	0.026	0.890
Erythema		0.185	0.327	0.249	0.184
Scales		0.263	0.161	0.263	0.160
Itching		0.436	0.016	0.106	0.576

*BSA* body surface area, *r* Spearman’s rho correlation coefficient, *P* > 0.05 = not significant, *P* < 0.05 = significant—*P* < 0.01 highly significant

## Discussion/conclusion

Mycosis fungoides is a form of dysregulated proliferation of CD4 T cells in the skin. The definitive etiology of MF is unknown, and multiple factors interplay in its pathogenesis. Association with infectious agents besides genetic and immunological factors was postulated [22]

Besides its role in regulating T cell development, SOCS-3 is one of the proteins expressed in malignant cells of CTCL cell lines. Still, its exact role played in the disease has not yet been thoroughly investigated [2]. This study aimed to assess the level of SOCS-3 in the lesional and perilesional skin of mycosis fungoides patients and compare it to normal healthy controls in a trial to evaluate the possible role of SOCS-3 in the pathogenesis of mycosis fungoides.

This study was conducted on 60 subjects (30 patch stage mycosis fungoides patients and 30 age and sex-matched normal healthy controls). Most of the patients were in stage Ib, and there was a female predominance in patients’ group; 66.7% of the patients were females, and 33.3% were males, with a ratio (2:1). Similar findings were reported by Amorim et al., where most of the early MF patients were in stage Ib but with a male to female ratio (1: 0.8). [23].

Levels of SOCS-3 in the control group ranged between 31.00 and 62.00 ng/mg(protein) with a mean value of 40.72 ± 7.80. Healthy normal controls were chosen to assess SOCS-3 levels in normal skin to reference our study and future studies. Also, it is not known whether there is an altered SOCS-3 expression in the normal-appearing skin of MF patients as there are no prior studies done to assess SOCS-3 level, but through Immunohistochemistry studies, SOCS-3 proteins were highly expressed in the epidermis of allergic contact dermatitis and psoriasis with relatively lower expression in atopic dermatitis (AD) skin but overall higher than non-lesional skin and normal healthy control skin [15, 16]. In the patients’ group, the mean value of the levels of SOCS-3 in lesional skin was 120.44 ± 15.24 ng/mg while it was 66.42 ± 17.98 ng/mg in perilesional skin. Upon comparing the mean values of the level of SOCS-3 in lesional, perilesional skin in patients and that in the control group, there was a statistically significant difference with a higher level in lesional than in perilesional skin, and both were higher than the SOCS-3 level in controls (*P* < 0.001). This shed light on the role that SOCS-3 could play in the pathogenesis of MF.

Upon comparison of SOCS-3 level in both lesional and perilesional skin as regards TNM staging in patients’ group, there was a significant difference between them with a higher level in lesional than perilesional skin whether stage Ia, Ib, or IIa (*P* < 0.001, *P* < 0.001, *P* < 0.001, respectively) that further adds a proof for the role of SOCS-3 in the well-established lesion of MF disease and this could propose a new modality for the treatment of MF if we try to counteract SOCS-3 effect. This also answers whether SOCS-3 level could be used as a marker for the severity of MF.

During mycosis fugoides progression, the expression of Th2 markers (e.g., GATA-3) and cytokines (e.g., IL-4, IL-5, and IL-10) increases, whereas the expression of Th1 transcription factors, such as T-cell-specific T-box transcription factor (T-bet), IFN-γ, STAT4, and IL-12 decreases [24]. What shifts the balance in favor of tumor progression remains to be largely unknown in CTCL. We hypothesize that SOCS 3 is one of the factors that aid in this shift and from early stage to advanced-stage MF.

SOCS3 regulates the activation and differentiation of naive CD4 + T cells, preferentially promoting Th2 and inhibiting Th1 differentiation through the inhibition of IL-12-mediated STAT4 activation. The overexpression of SOCS1 and SOCS3 in T cells inhibits IFN-α-induced phosphorylated STAT1 and the transcription of IFN-stimulated genes [25].

Unlike the T-helper cells, Tregs are deficient in SOCS3 expression. The in vitro overexpression of SOCS3 in Tregs decreases their proliferation and Foxp3 expression. Similarly, SOCS3 removal in T-lymphocytes upregulates CTLA-4 expression, which shows that SOCS3 negatively regulates CTLA-4 level in T cells and provides a mechanistic explanation for the expansion of Tregs in advanced-stage MF [26].

After a detailed literature review, this was the first study that assessed the level of SOCS-3 in MF quantitively. There was no statistical correlation between SOCS-3 level in patients’ group, whether lesional or perilesional, and demographic data regarding their age and sex; (*P* > 0.05, 0.05, respectively), or clinical parameters, namely duration BSA, erythema, and scaling. This further proved the role played by SOCS-3 in pathogenesis in MF without being influenced by any demographic data or disease parameters.

On the other hand, there was a positive correlation between itching and lesional SOCS-3 level. This could point to the role of SOCS-3 in the induction of itching in MF that is one of the important distressing symptoms that affect the quality of life of MF patients. Sometimes, it is the only presenting symptom as in the invisible variant of MF.

The pathogenesis of itching in MF is not entirely understood. The fact that antihistaminic is poorly effective in relieving MF pruritis and increased number of eosinophils, but not mast cells infiltrating skin in patients with marked itching, suggests an altered mechanism of itching may be related to cytokine profile of malignant T cells and other immune cells [27, 28]. During the early stages, MF is characterized by Th1 response, and as the disease progresses, Th2, with its signature cytokines dominates [29]. Different mediators are implicated in pathogenesis as IL6, IL13, IL31, IL2, IL4, IL5, IL10, substance P, vascular endothelial growth factor (VEGF), and nerve growth factor (NGF) [27, 30, 31].

The grade of itching was related to an increased level of eosinophils rather than mast cells in MF. Interleukin 31 secreted from eosinophils and Th2 cells is considered one of the itch mediators, and its level correlates with itching severity [28, 30]. It was noted that IL31 is an important SOCS-3 inducer as a negative feedback mechanism [32].

Other cytokines, e.g., IL10 and IL13, were found to enhance SOCS-3 expression by activating STAT6 [33].On the other hand, SOCS-3 promotes Th 2 cells development that secretes IL13 [34]. In diseases with dominant Th2, the levels of SOCS-3 in peripheral T-cells correlated with disease severity and IgE levels [35].

From these observations, SOCS-3 can induce or influence the itching symptom in MF. IL10, and IL31 increase SOCS-3 expression that can worsen MF lesions by upregulation of Th2. This can increase the production of IL4,5, 10, 13 that mediate itching. Also, Th2 can stimulate antibody production (including IgE) from B cells that would further intensify itching.

In conclusion, these data are preliminary, and more studies are needed to clarify the exact role of SOCS-3 in MF and other types of cancers given its complex action as a tumor progressor in some cases and its antitumor effect in others, making it a promising therapeutic target.

## Data Availability

The data that support the findings of this study are available on request from the corresponding author.
